# Estimation of influenza incidence and analysis of epidemic characteristics from 2009 to 2022 in Zhejiang Province, China

**DOI:** 10.3389/fpubh.2023.1154944

**Published:** 2023-05-18

**Authors:** Haocheng Wu, Ming Xue, Chen Wu, Zheyuan Ding, Xinyi Wang, Tianyin Fu, Ke Yang, Junfen Lin, Qinbao Lu

**Affiliations:** ^1^Center for Disease Control and Prevention (Zhejiang CDC), Zhejiang, Hangzhou, China; ^2^Hangzhou Center for Disease Control and Prevention (HZCDC), Hangzhou, China

**Keywords:** estimation, influenza, random forest, moving epidemic method, Joinpoint regression

## Abstract

**Background:**

Influenza infection causes a huge burden every year, affecting approximately 8% of adults and approximately 25% of children and resulting in approximately 400,000 respiratory deaths worldwide. However, based on the number of reported influenza cases, the actual prevalence of influenza may be greatly underestimated. The purpose of this study was to estimate the incidence rate of influenza and determine the true epidemiological characteristics of this virus.

**Methods:**

The number of influenza cases and the prevalence of ILIs among outpatients in Zhejiang Province were obtained from the China Disease Control and Prevention Information System. Specimens were sampled from some cases and sent to laboratories for influenza nucleic acid testing. Random forest was used to establish an influenza estimation model based on the influenza-positive rate and the percentage of ILIs among outpatients. Furthermore, the moving epidemic method (MEM) was applied to calculate the epidemic threshold for different intensity levels. Joinpoint regression analysis was used to identify the annual change in influenza incidence. The seasonal trends of influenza were detected by wavelet analysis.

**Results:**

From 2009 to 2021, a total of 990,016 influenza cases and 8 deaths were reported in Zhejiang Province. The numbers of estimated influenza cases from 2009 to 2018 were 743,449, 47,635, 89,026, 132,647, 69,218, 190,099, 204,606, 190,763, 267,168 and 364,809, respectively. The total number of estimated influenza cases is 12.11 times the number of reported cases. The APC of the estimated annual incidence rate was 23.33 (95% CI: 13.2 to 34.4) from 2011 to 2019, indicating a constant increasing trend. The intensity levels of the estimated incidence from the epidemic threshold to the very high-intensity threshold were 18.94 cases per 100,000, 24.14 cases per 100,000, 141.55 cases per 100,000, and 309.34 cases per 100,000, respectively. From the first week of 2009 to the 39th week of 2022, there were a total of 81 weeks of epidemics: the epidemic period reached a high intensity in 2 weeks, the epidemic period was at a moderate intensity in 75 weeks, and the epidemic period was at a low intensity in 2 weeks. The average power was significant on the 1-year scale, semiannual scale, and 115-week scale, and the average power of the first two cycles was significantly higher than that of the other cycles. In the period from the 20th week to the 35th week, the Pearson correlation coefficients between the time series of influenza onset and the positive rate of pathogens, including A(H3N2), A (H1N1)pdm2009, B(Victoria) and B(Yamagata), were − 0.089 (*p* = 0.021), 0.497 (*p* < 0.001), −0.062 (*p* = 0.109) and − 0.084 (*p* = 0.029), respectively. In the period from the 36th week of the first year to the 19th week of the next year, the Pearson correlation coefficients between the time series of influenza onset and the positive rate of pathogens, including A(H3N2), A (H1N1)pdm2009, B(Victoria) and B(Yamagata), were 0.516 (*p* < 0.001), 0.148 (*p* < 0.001), 0.292 (*p* < 0.001) and 0.271 (*p* < 0.001), respectively.

**Conclusion:**

The disease burden of influenza has been seriously underestimated in the past. An appropriate method for estimating the incidence rate of influenza may be to comprehensively consider the influenza-positive rate as well as the percentage of ILIs among outpatients. The intensity level of the estimated incidence from the epidemic threshold to the very high-intensity threshold was calculated, thus yielding a quantitative standard for judging the influenza prevalence level in the future. The incidence of influenza showed semi-annual peaks in Zhejiang Province, including a main peak from December to January of the next year followed by a peak in summer. Furthermore, the driving factors of the influenza peaks were preliminarily explored. While the peak in summer was mainly driven by pathogens of A(H3N2), the peak in winter was alternately driven by various pathogens. Our research suggests that the government urgently needs to address barriers to vaccination and actively promote vaccines through primary care providers.

## Background

Influenza is an acute respiratory infectious disease caused by influenza virus, which is an RNA virus that is divided into three types: A, B and C (1, 2). The types of A, B and C not only reflect the order in which the virus was discovered but also, more importantly, reflect the order of harm to human beings (3, 4). Influenza A virus is the main epidemic strain and can lead to a global influenza pandemic. It is widely transmitted among animals, which can lead to a flu epidemic among animals and cause a large number of animal deaths (5, 6). Compared with influenza A virus, influenza B virus causes only local outbreaks and does not cause pandemics. Influenza C virus generally appears in a scattered form; it mainly affects infants and does not cause epidemics (7). Subtypes of influenza A–i.e., H3N2 virus and H1N1pdm2009 virus–and influenza B virus are the main viruses circulating in the population (8).

Influenza is mainly transmitted by droplets, and people are generally susceptible to infection (2). Influenza virus can cause different degrees of infection, ranging from mild illness requiring hospitalization to severe illness and sometimes even death (9, 10). Each year, a substantial disease burden is attributed to seasonal influenza (11). Influenza infections annually affect approximately 8% of adults and approximately 25% of children, resulting in approximately 400,000 respiratory-related deaths worldwide according to the World Health Organization (WHO) (12). From 2006 to 2019, the annual number of outpatient visits for influenza-related influenza-like diseases (ILIs), number of hospitalizations for severe acute respiratory infections (SARIs) and number of excessive respiratory deaths in mainland China were 3 million, 2.34 million and 90,000,000, respectively, leading to a total economic burden of 26.38 billion yuan and accounting for 0.266 ‰ of the 2019 GDP (13). However, compared with the number of reported cases of influenza, the actual prevalence of influenza may be greatly underestimated (14). From 2005 to 2010, the number of reported influenza cases in mainland China was only 45,672, 57,557, 36,434, 41,692, 198,381 and 64,502, respectively; however, the estimated incidence of influenza in Guangzhou city in 2006 was 2,382/100,000, approximately equal to 237,413 cases, which was far more than the reported number of cases in the whole country (15). Therefore, reports of influenza-like illness (ILIs) are usually used to estimate the trend of disease instead of reports of influenza alone (14, 16). However, according to a previous study, the specificity of ILIs for estimating the incidence of influenza is only 77%, thus leading to an overestimate of the incidence of influenza (14). The purpose of this study is to establish an influenza estimation model based on the percentage of ILIs among outpatients and the influenza-positive rate and to correct the reported incidence level. Furthermore, the moving epidemic method (MEM) was applied to calculate the epidemic threshold for different intensity levels based on the estimated weekly incidence. Joinpoint regression analysis was used to identify the annual change in estimated influenza incidence. The seasonal periodicity of weekly incidence of influenza was detected by wavelet analysis.

## Materials and methods

### Data collection

Data regarding newly diagnosed influenza cases and the prevalence of ILIs among outpatients in Zhejiang Province were collected between week 1 in 2009 and week 39 in 2022 from the China Disease Control and Prevention Information System. The population data used to calculate the incidence rate was updated by the company responsible for system operation and maintenance and the new population data was imported into the system every December. The incidence rate of influenza was computed by the system and can be exported. Data on the influenza A (H1N1)pdm2009 subtype from 2009 to 2013 were reported separately, and these cases were added to the total number of influenza cases in the corresponding year. Specimens were sampled from some cases and sent to laboratories for A(H3N2), A (H1N1)pdm2009, B(Victoria) and B(Yamagata) influenza nucleic acid testing or antigen testing. The diagnosis of influenza virus is based on the diagnosis and treatment criteria, including ‘Diagnostic criteria for influenza’ (Version 2008), ‘Guidelines for diagnosis and treatment of influenza A (H1N1)pdm2009’ (3rd Edn 2009) and ‘Guidelines for diagnosis and treatment of influenza’ (Version 2019) (17–19). When a symptomatic case has positive results of any of the following pathogenic tests, it is diagnosed as a confirmed case, including positive influenza virus nucleic acid test, positive influenza antigen test, positive influenza virus culture isolation, and the level of influenza virus-specific IgG antibody in the double sera of acute and convalescent patients increased by 4 times or more.

### Random forest analysis

Random forest is a widely used method for data prediction and classification calculation. Random forest is a combinatorial classification intelligent algorithm based on statistical learning theory. The basic idea of this method is to combine multiple weak classifiers with complementary functions to form a strong classifier. By reducing the impact of single classifier errors, the accuracy and stability of model classification can be improved. The main step is to randomly select k subtraining sample sets from the total training sample set through bootstrap sampling and establish a decision classification subtree model. Then, m is randomly selected from the n indices of each node in the classification tree and segment according to the optimal segmentation index. The previous step is repeated to traverse K classification subtrees to determine multiple classification results. Then, the final classification result is determined by voting. Approximately 36.8% of the samples in this model will not appear in the bootstrap sampling set. This part of the data is called OOB (Out Of Bag) data. OOB data can be used to evaluate the decision subtree model and determine the error classification rate of the decision subtree, namely, the OOB error (20).

We established the training model based on the reported influenza cases as a dependent variable and the observed weekly percentage of ILIs among outpatients and influenza-positive rate during 2019–2022 as the independent variables. In the next step, we estimated the weekly number of influenza cases from 2009 to 2018.

### Joinpoint regression

Joinpoint regression is also called piecewise regression, broken-line regression or multiphase regression. This model does not require the data series itself to show an obvious trend, and it is increasingly used to determine the degree of change in time series data. Joinpoint regression analysis software uses the *Z* score to test the hypothesis of segmentation points to determine whether the data have sufficient evidence to add how many segmentation points. The first step assumes that there is no segmentation point, that is, *H_0_*. If *H_0_* is rejected, then the analysis is used to test whether there is statistical significance between 1 segmentation point and n segmentation points, and so on (21).

The objective indicator was the annual percent change (APC) of each period segment, estimated according to the following formula:


(1) $$APCi=\left[\exp\left(\beta_{i}\right)-1\right]\times 100,$$

where $\beta_{i}$ represents the slope of the period segment (22).

### Wavelet analysis (22)

The wavelet method is a reasonable method for studying periodic phenomena in time series, especially when the existence of potential frequency changes with time. Morlet wavelet is used to analyze the frequency structure of univariate and bivariate time series. This continuous complex wavelet leads to the continuous complex wavelet transform of the time series at hand, so the information can be saved by carefully selecting the time and frequency resolution parameters. The transformation can be divided into a real part and an imaginary part to provide information about the local amplitude and instantaneous phase of any periodic process in time, which is a prerequisite for studying the correlation between two time series (22).

### Moving epidemic method (23)

The MEM includes three main steps. The first step is to determine the time length of the epidemic season and the time nodes of the beginning and end of the epidemic season from a professional perspective based on the epidemic law of the disease and to divide the epidemic season into the pre-epidemic period (from the beginning of the epidemic season to the end of the epidemic season), the epidemic period (from the beginning of the epidemic season to the end of the epidemic season) and the post-epidemic period (from the end of the epidemic season to the end of the epidemic season). The second step is to calculate the pre-epidemic baseline, pre-epidemic threshold (epidemic start threshold), post-epidemic baseline and post-epidemic threshold (epidemic end threshold) by using the pre-epidemic and post-epidemic monitoring index values of historical data. The pre-epidemic/post-epidemic baseline is calculated using the arithmetic mean of all its monitoring indicators. For the calculation of the pre-epidemic/post-epidemic threshold of the current epidemic season, the n maximum monitoring indicators (*n* = 30/N, N is the number of epidemic seasons) of each historical epidemic season are taken, for a total of *n* × *N* = 30 values, and the upper limit of its one-sided 95% confidence interval is calculated. The third step is to calculate the different-intensity thresholds of the current epidemic period by using the monitoring index values of the epidemic period in the historical epidemic season for monitoring and warning. The specific method is as follows: select the maximum value of n monitoring indicators in the historical epidemic period, totaling *n* × *N* = 30 values; then, define the upper limit of the one-sided 40, 90 and 97.5% confidence intervals of the geometric mean of the 30 maximum monitoring index values, which correspond with the medium, high and extremely high intensity thresholds, respectively. Influenza epidemic intensity level is defined as ① baseline: weekly monitoring index value < epidemic start/end threshold; ② low-intensity epidemic: epidemic threshold ≤ weekly monitoring index value < medium-intensity threshold; ③ moderate-intensity epidemic: moderate-intensity threshold ≤ weekly monitoring index value < high-intensity threshold; ④ high-intensity epidemic: high-intensity threshold ≤ weekly monitoring index value < extremely high-intensity threshold; ⑤ extremely high-intensity epidemic: weekly monitoring index value ≥ extremely high-intensity threshold.

### Statistical analysis

The joinpoint regression model was constructed using joinpoint software (version 4.5.0.1). The random forest modeling, MEM model and wavelet analysis were run by R Studio (version 1.2.5001). A *p* value less than 0.05 indicated statistical significance for all the tests.

## Results

### Basic information

**Figure 1:**
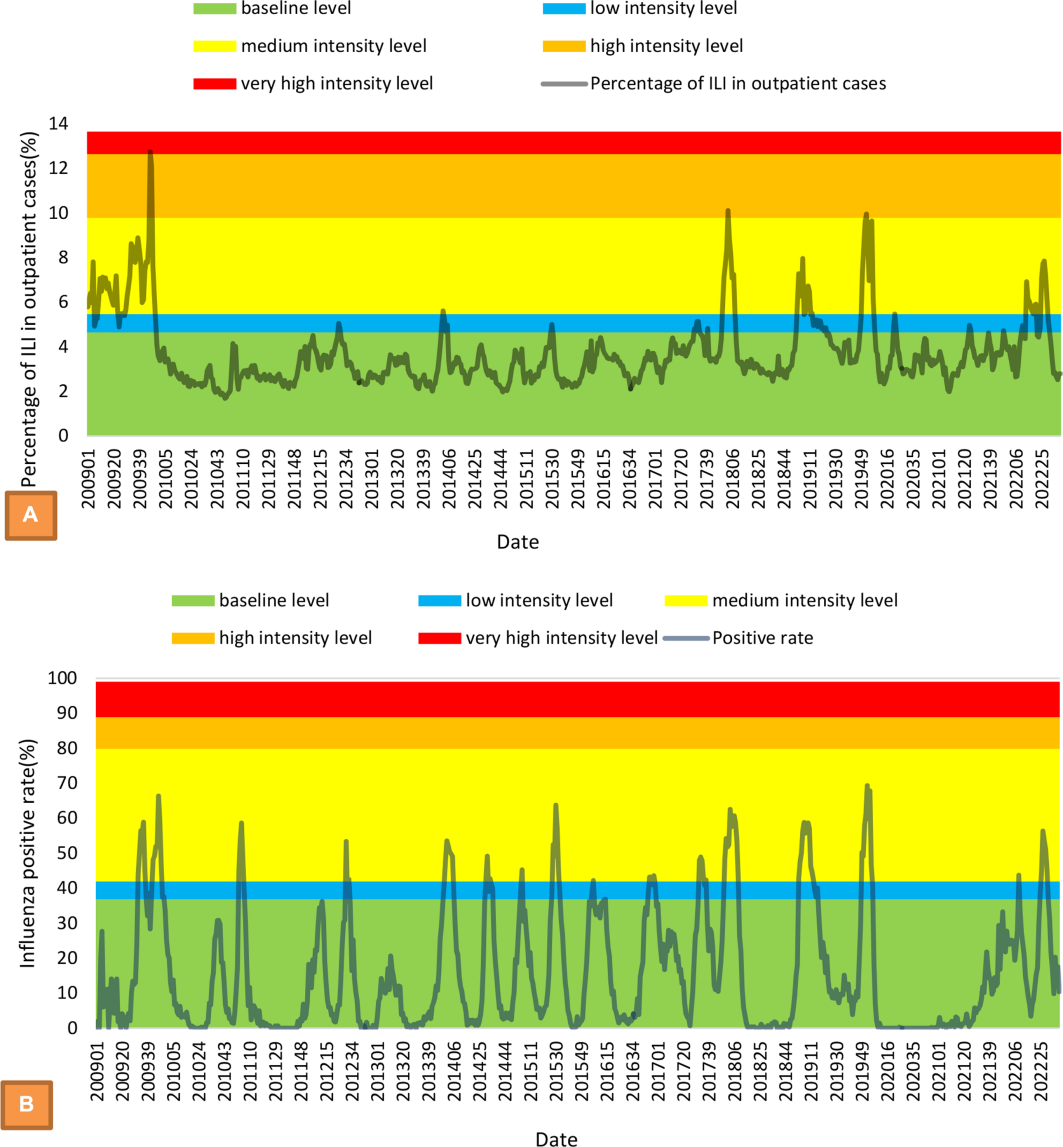
MEM model with intensity levels and time-series of weekly percentage of ILIs among outpatients **(A)** and weekly influenza-positive rate **(B)**.

From 2009 to 2021, a total of 990,016 influenza cases and 8 related deaths were reported in Zhejiang Province. The annual influenza incidence varies widely from 4.9498 cases per 100,000 to 850.2056 cases per 100,000. The percentage of ILIs among outpatients fluctuated across years and followed a bimodal seasonal pattern, where the peak epidemic period was always from the 51st week of a year to the 8th week of the next year; additionally, there was sometimes a small peak in summer. The highest prevalence of ILIs among outpatients was 12.11%, which was observed in the 48th week of 2009 and was mainly affected by the influenza A (H1N1)pdm2009 subtype. The lowest prevalence was 1.69%, which was observed in the 49th week of 2010 (Figure 1A). The intensity levels of the prevalence of ILIs among outpatients from the epidemic threshold to the very high-intensity threshold were 4.66, 5.48, 9.79, and 12.65%, respectively. The weekly influenza-positive rate was similar across different years, and the peak of the epidemic was driven by the alternation of different influenza subtypes into dominant strains. The lowest weekly influenza-positive rate was 0%, and the highest rate was 69.33%, which was observed in the first week of 2020 (Figure 1B). The intensity levels of the influenza-positive rate from the epidemic threshold to the very high-intensity threshold were 37.01, 42.23, 80.33 and 89.64%, respectively.

### Estimation of the incidence of influenza and the trend from 2009 to 2021

**Figure 2:**
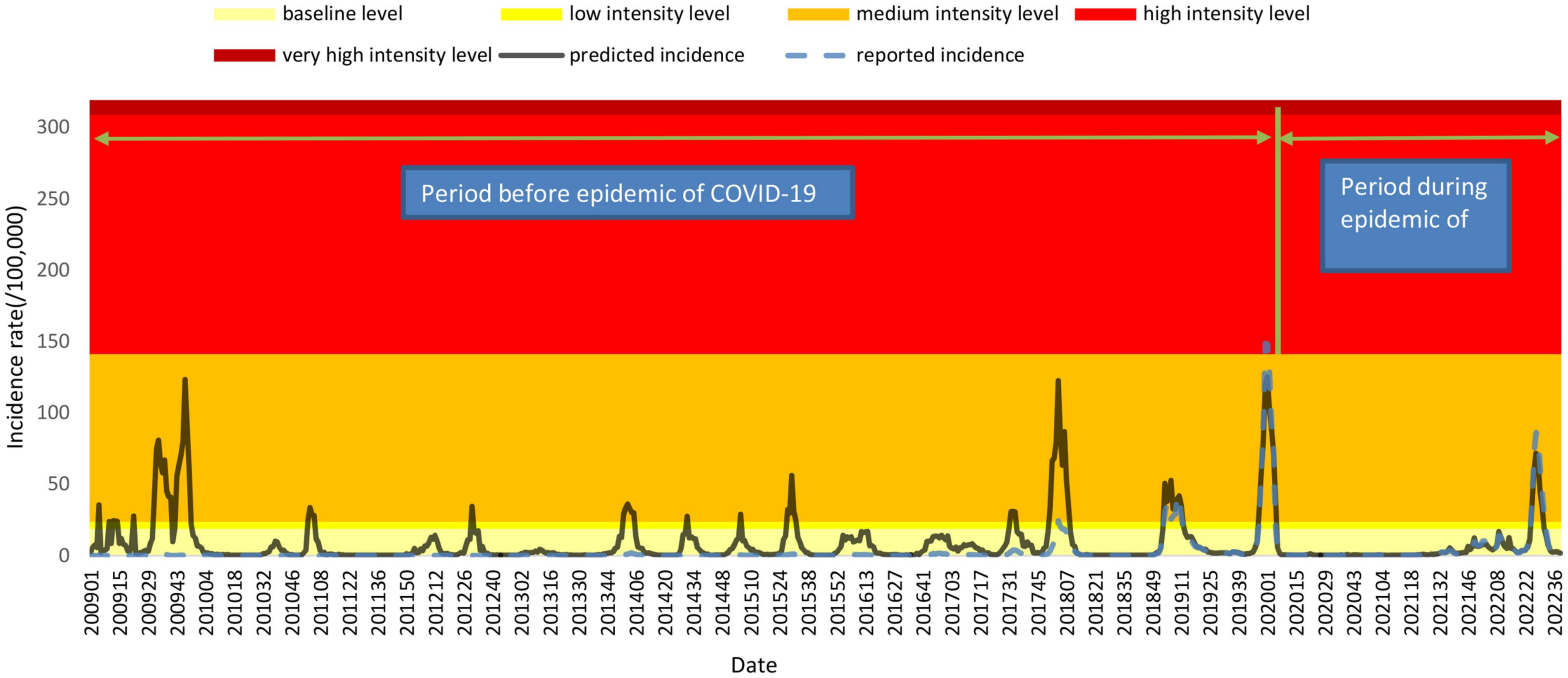
MEM model with intensity levels and time series of weekly estimated incidence and reported incidence.

Based on the observed weekly percentage of ILIs among outpatients and the influenza-positive rate during 2019–2022, the training model was established. The mean absolute percentage error (MAPE) of the model was 26.10%. The raw predicted data and the actual data were well matched (Figure 2). The numbers of estimated influenza cases from 2009 to 2018 were 743,449, 47,635, 89,026, 132,647, 69,218, 190,099, 204,606, 190,763, 267,168 and 364,809, respectively. However, the number of reported cases during the same periods was only 20,385, 4,063, 2,694, 2,908, 3,302, 9,700, 7,970, 14,394, 30,434 and 94,091, respectively. The total number of estimated influenza cases is 12.11 times the number of reported cases.

**Figure 3:**
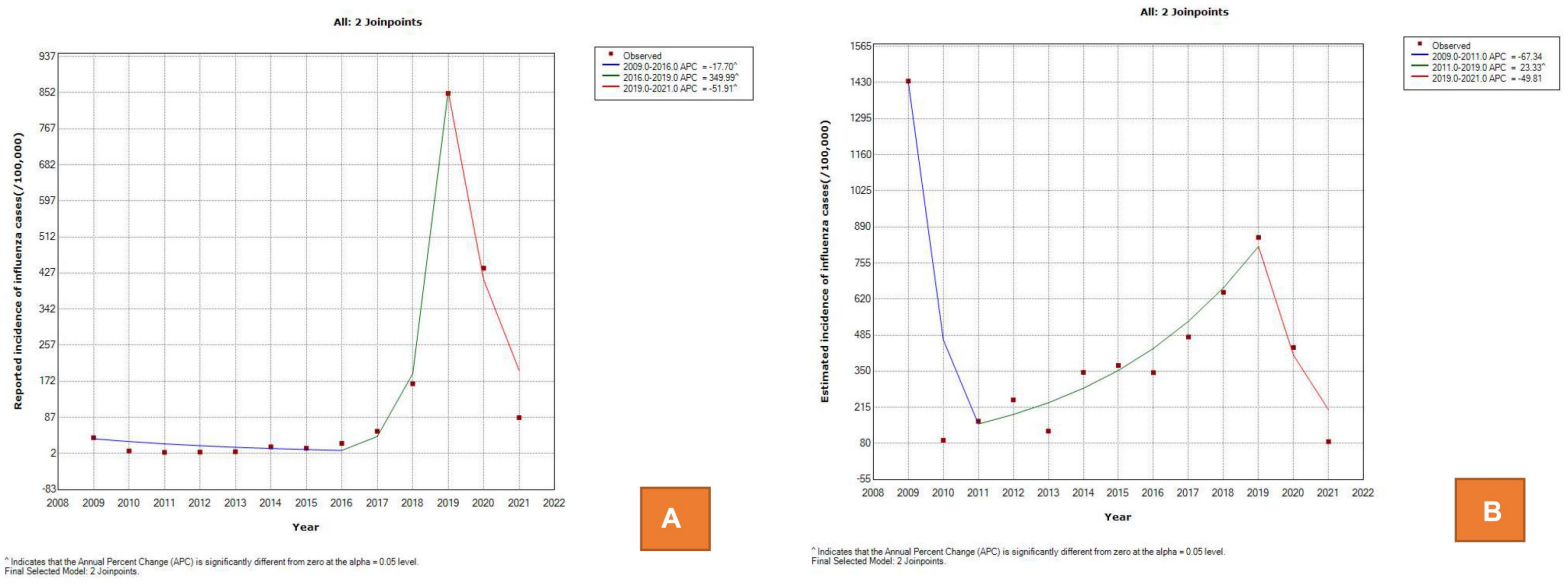
Trend of influenza incidence between 2009 and 2021 in the joinpoint regression model. **(A)** The reported annual incidence rate. **(B)** The estimated annual incidence rate.

The time series of the estimated annual incidence rate is significantly different from the curve of the reported annual incidence rate. The final selected model of the reported annual incidence rate was the 2 joinpoints relative to the 0 joinpoint (*p* < 0.001) and 1 joinpoint (*p* < 0.001). The APCs were − 17.70 (95% CI: −31.40 to −1.20) from 2009 to 2016, 349.99 (95% CI: 206.40 to 560.80) from 2016 to 2019, and − 51.91 (95% CI: −62.00 to −39.20) from 2019 to 2021, which were significant differences at the 0.05 level (test statistic = −2.70, 10.10, −8.00, respectively, *p* < 0.001). The APC indicated a gradual decreasing trend from 2009 to 2016; then, it showed a sharp upward trend from 2016 to 2019 and decreased again after 2019 (Figure 3A). The final selected model of the estimated annual incidence rate was also the 2 joinpoints relative to the 0 joinpoint (*p* < 0.001) and 1 joinpoint (*p* = 0.01). The APC was 23.33 (95% CI: 13.2 to 34.4) from 2011 to 2019, indicating a monotonically increasing trend, but the APC before 2011 and after 2019 was not significantly different from zero(Figure 3B).

### Intensity level of influenza, seasonal periodicity and driving factors

The inner parameters of the model was set from 1.5 to 3%, increasing by 0.1% each time, and the fitted Youden index of all models was between 0.4709 and 0.5450, of which the parameter corresponding to the maximum Youden index is 1.8%, and the correspondingspecificity, sensitivity, positive predictive value and negative predictive value, were 0.95, 0.56, 0.75 and 0.89, respectively. The intensity levels of the estimated incidence from the epidemic threshold to the very high-intensity threshold were 18.94 cases per 100,000, 24.14 cases per 100,000, 141.55 cases per 100,000, and 309.34 cases per 100,000, respectively. According to the estimated time series of influenza incidence, the influenza incidence level is at the baseline level in most of the time periods, and most of the peaks of the influenza epidemic corresponded to a the moderate intensity level. From the first week of 2009 to the 39th week of 2022, there were a total of 81 weeks of epidemics, of which the epidemic period reached a high intensity in 2 weeks, the epidemic period was at a moderate intensity in 75 weeks, and the epidemic period was at a low intensity in 2 weeks. The epidemic reached a high intensity in the 52nd week of 2019 and the first week of 2020; the respective incidence rates were 148.89 cases per 100,000 and 148.20 cases per 100,000. Based on the reported incidence, the number of influenza cases reported in the week before 2018 was very low and fluctuated very little between different weeks, making it impossible to detect the peak epidemic (Figure 2).

**Figure 4:**
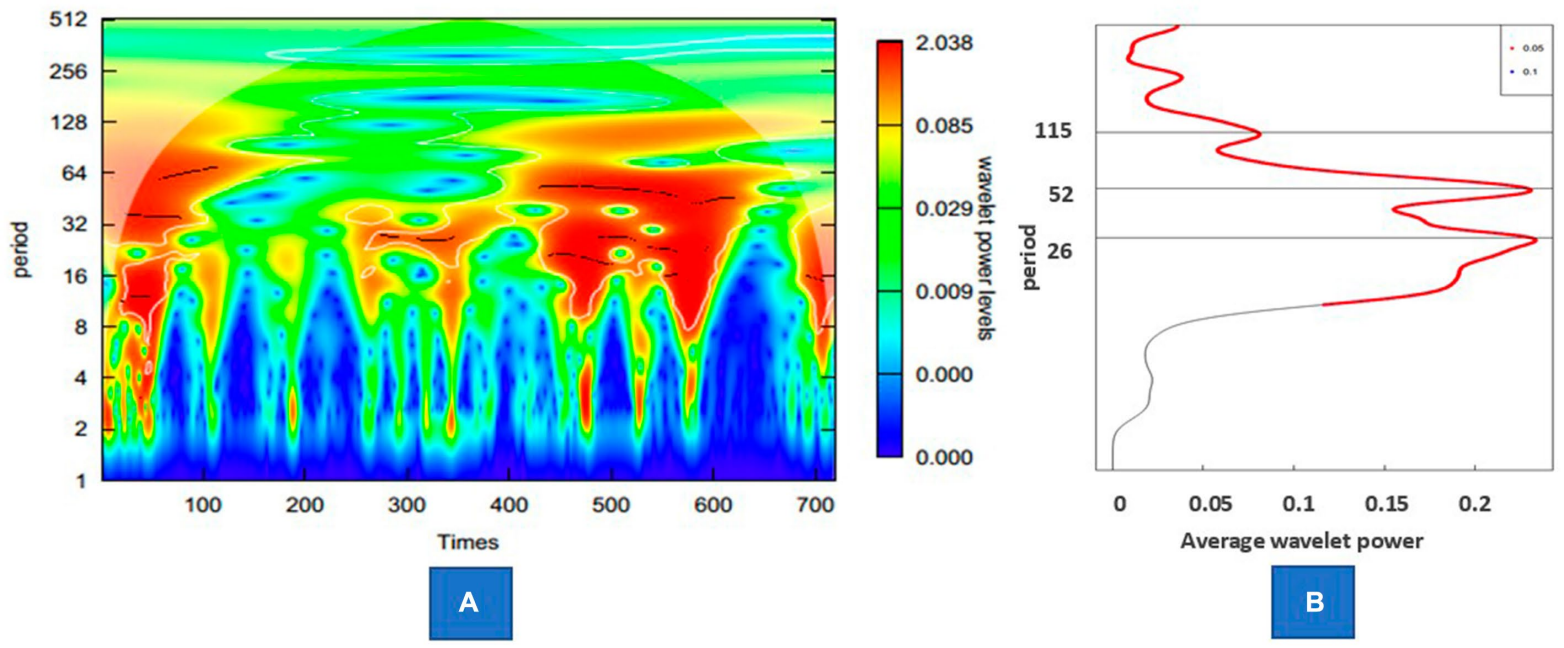
Wavelet power of the estimated incidence of influenza. **(A)** Wavelet power spectrum of the series. **(B)** The average power of the whole period.

For the incidence rate of reported cases, it is almost impossible to observe obvious seasonal characteristics before 2017. After adjusting the reported incidence rate, an obvious peak of incidence in winter and spring can be observed, which usually occurs from December to March of the next year from 2009 to 2022. In addition, there was also a peak incidence in summer in 2009, 2014, 2015, 2017 and 2022. Figure 4 shows the wavelet power spectrum for the estimated incidence of influenza. The average power was significant on the semiannual scale, 1-year scale, and 115-week scale, and the average power of the first two cycles was significantly higher than that of the other cycles. The average power indicated that this disease showed half a year peaks, including a main peak from December to January of the next year followed by a peak in summer. This periodicity is not significant at all times. From the 47th week of 2010 to the 40th week of 2013 and from the 8th week of 2020 to the 32nd week of 2021, there was no obvious semiannual periodicity of the incidence. The 1-year scale was also relatively weak from the 44th week of 2011 to the 41st week of 2013.

Because the influenza season has two waves, it was divided into two periods: the first section is from the 20th week to the 35th week, and the second section is from the 36th week of the first year to the 19th week of the next year. In the first period, the Pearson correlation coefficients between the time series of influenza onset and the positive rate of pathogens, including A(H3N2), A (H1N1)pdm2009, B(Victoria) and B(Yamagata), were 0.497 (*p < 0.001*), −0.089 (*p = 0.021*), −0.062 (*p = 0.109*) and − 0.084 (*p = 0.029*), respectively. In the second period, the Pearson correlation coefficients between the time series of influenza onset and the positive rate of pathogens, including A (H1N1)pdm2009, A (H3N2), B (Victoria) and B (Yamagata), were 0.516 (*p < 0.001*), 0.148 (*p < 0.001*), 0.292 (*p < 0.001*) and 0.271 (*p < 0.001*), respectively.

## Discussion

Influenza virus infections are very common worldwide, and the incidence of influenza can only be estimated (14, 24). Two major surveillance subsystems under the China Disease Control and Prevention Information System, including the Infectious Disease Monitoring and Reporting System and the Influenza-Like Illness (ILI) Surveillance System, have been officially used for the monitoring and analysis of influenza in China (25). However, due to the limitations of diagnostic criteria and the fact that not everyone was tested for influenza, the reported incidence of influenza differs greatly from the actual incidence level (14, 15). Therefore, influenza-like illness (ILI) is usually used to estimate the trend of disease instead of influenza (14, 16). However, a previous study showed that the level of influenza-like cases is important for monitoring influenza infection and had the best sensitivity (86%) and specificity (77%) values (26). However, based on the percentage of ILIs among outpatients, the actual prevalence of influenza may be overestimated. In our study, from the 11th week of 2020 to the 53rd week of 2020, the weekly median level of the percentage of ILIs among outpatients was 3.21, which was higher than the weekly median level from 2010 to 2016; however, the weekly average level of influenza-positive rate in the same period was almost 0. This suggests that the level of influenza incidence during this period is very low, and the use of the percentage of ILIs among outpatients instead of measuring influenza cases will lead to significant overestimation. Therefore, an appropriate method for estimating the incidence rate of influenza may be to comprehensive consider the influenza-positive rate in addition to the percentage of ILIs among outpatients.

According to our research, the actual incidence level of influenza is far higher than the current reported number, which also indicates that the disease burden of influenza has been seriously underestimated in the past. The reported incidence of influenza increased rapidly in 2019, mainly due to the revision of the Guidelines for the Diagnosis and Treatment of Influenza, in which the rapid antigen detection method was added as the diagnostic standard. Compared with previous studies, only a few samples were reported with positive nucleic acid detection. According to the number of reported cases, the incidence of influenza from 2011 to 2016 showed a downward trend, but after estimation with the model, we found that the incidence of influenza in this period actually showed an upward trend, which suggested that incomplete diagnosis and inaccurate reporting would lead to misunderstanding of the epidemic trend. The result of a rapid increasing trend of influenza is similar to several previous studies (25, 27). There are several reasons that may be related to the increasing trend of influenza during this period. Influenza vaccination is the most effective way to prevent influenza infection and reduce severe influenza-related complications (28). The reductions in the numbers of vaccine supplements and low vaccination coverage rate might be an important factor for the increased incidence of influenza. The lack of an influenza vaccine may be mainly due to the vaccine scandal caused by improper vaccine storage and production in 2016 and 2018, respectively (25, 29). In addition, the use of automatic data acquisition and reporting systems, which improved both the quantity and quality of data collection, might be another reason (30). In addition, the rapid increase in the number of airlines and high-speed rail transport in recent years will make it easier for influenza virus to spread on a larger scale and in a shorter time across the country (31). The outbreak growth of influenza cases in 2009 is obviously attributable to the spread and widespread impact of influenza A (H1N1) pdm2009 (25). Due to the response to the COVID-19 pandemic, some prevention and control measures, including the improvement of self-protection, the isolation of cases and close contacts, and the reduction in social activities of the population, have led to a reduction in several infectious diseases, including influenza and tuberculosis transmission, in 2020, and this effect may last until 2021 (8, 32). During the period of this study, Chinese Mainland adopted the strategy of containment and elimination of the COVID-19 epidemic. Once cases with suspicious symptoms were found, nucleic acid testing and strict diagnosis were required, and all possible close contacts were tracked and managed to achieve the goal of clearing cases in a short period of time. Therefore, the context of each case of COVID-19 is very clear, and there will be no misclassification with influenza cases. According to the model of the estimated annual incidence rate, the decreasing trend was not significantly different from zero after 2019, which suggests that influenza incidence is gradually returning to the level before 2020 (8).

The epidemic threshold is affected by the regional heterogeneity of monitoring data, and the intensity thresholds vary according to the historical rates (33). Through the correction of the influenza incidence level, we calculated the influenza incidence intensity grading threshold, which provides a quantitative standard for judging the influenza prevalence level in the future. Before the winter peak in 2019–2020, the epidemic level of influenza in Zhejiang Province was at the middle or lower level. Influenced by the prevention and control of COVID-19, the winter peak of 2019–2020 was interrupted, during which the influenza epidemic was at the baseline level. This impact lasted for nearly 2 years until the summer peak of influenza reappeared in 2022. In general, the established influenza grading model has good performance, especially its specificity, which reaches 95%. According to previous studies, compared with sensitivity, specificity is a more important indicator for detecting influenza epidemics because false-positives will cause excessive public concern and trigger unnecessary influenza prevention and control measures, such as antiviral use, enhanced vaccination or nondrug intervention (23).

According to wavelet analysis, the influenza epidemic in Zhejiang Province mainly experiences semiannual peaks – one in winter and the other in summer–while the periodicity of the long cycle across the year is relatively less significant, which is similar to previous studies (34, 35). Further research shows that the peak of influenza incidence in Zhejiang Province in summer was mainly driven by pathogens of A(H3N2), while the peak in winter was alternately driven by various pathogens, including A(H3N2),A (H1N1)pdm2009, B(Victoria) and B(Yamagata). However, the summer influenza epidemic peak in southern China may not always occur regularly (34). In our study, the summer epidemic was not obvious in two periods, and the latter period was mainly due to the decline in the overall incidence level caused by the management and control of the COVID-19 pandemic. Another reason for the summer epidemic is the impact of relative humidity (RH) on the survival and transmission of influenza virus (36). In summer, influenza activity is mainly driven by high humidity rather than high temperature because contact transmission might be predominant due to the increasingly large droplets produced in a high RH environment (37). Furthermore, the high incidence in winter is affected by many reasons, including the inhibition of mucociliary clearance, the low RH environment where aerosol transmission is predominant, the decreased activities of proteases and increased indoor crowding (3).

In conclusion, the total number of estimated influenza cases is 12.11 times the number of reported cases. The actual incidence level of influenza is far higher than the current reported number, which also indicates that the disease burden of influenza has been seriously underestimated in the past. An appropriate method for estimating the incidence rate of influenza may be to comprehensively consider the percentage of ILIs among outpatients as well as the influenza-positive rate. The APC was 23.33 (95% CI: 13.2 to 34.4) from 2011 to 2019, indicating a constant increasing trend during this period. The intensity level of the estimated incidence from the epidemic threshold to the very high-intensity threshold was calculated, which provides a quantitative standard for judging the influenza prevalence level in the future. The incidence of influenza showed half a year peaks, including a main peak from December to January of the next year followed by a peak in summer in Zhejiang Province. Furthermore. The driving factors of the influenza peak have been preliminarily explored, while the peak in summer was mainly driven by pathogens of A(H3N2), and the peak in winter was alternately driven by various pathogens. According to previous studies, the average annual influenza vaccination rate for the entire population in China was only 2%, while the vaccination rate for the old adult aged over 60 was 3.8%, the overall influenza vaccination rate in the Chinese population is still very low (38). As influenza vaccination is the most important measure of preventing influenza infections, our research suggests that the government urgently needs to address barriers to vaccination and actively promote vaccines through primary care providers (28).

## Limitations

There are several limitations in our research that need to be acknowledged. First, because pathogenic surveillance cannot be carried out in all cases, the positive samples only represent a portion of cases, which may lead to bias to some degree. Second, the influenza incidence data were reported from the case visit report system, which might be affected by many factors, such as the case visit rate, the type of medical institution, and the detection rate of influenza pathogens. In future research, it should be considered to include mild and asymptomatic cases without medical treatment as much as possible to obtain a more complete infection spectrum. For example, it is possible to actively detect close contacts in school cluster outbreaks, or use positive results from community residents’ self-testing to supplement infection spectrum data. Third, data on factors influencing the peak incidence of different influenza subtypes have not been collected, and the driving factors causing the conversion between subtypes have not been resolved. Fourth, due to inaccurate classification of causes of death, the actual number of deaths caused by influenza cannot be estimated and the case fatality rate of influenza is significantly underestimated. Fifth, the model established in this study is black box, so the specific relationship between the incidence rate of influenza and the predicted independent variable is still unclear.

## Data availability statement

The raw data supporting the conclusions of this article will be made available by the authors, without undue reservation.

## Ethics statement

The studies involving human participants were reviewed and approved by the Ethics Committee of the Zhejiang Provincial Centers for Disease Control and Prevention. Written informed consent to participate in this study was provided by the participants’ legal guardian/next of kin.

## Author contributions

JL and HW: conceived and designed the research. QL, ZD, CW, XW, TF, and KY: data collection. HW: data analysis and wrote the paper. JL and MX: reviewed and revised the paper. All authors read and approved the final manuscript.

## Funding

This work was supported by the Major Science and Technology Project of the Science and Technology Department of Zhejiang Province (2021C03038, 2022C03109, 2022C03183). The funders had no role in study design, data collection and analysis, decision to publish, or preparation of the manuscript.

## Conflict of interest

The authors declare that the research was conducted in the absence of any commercial or financial relationships that could be construed as a potential conflict of interest.

## Publisher’s note

All claims expressed in this article are solely those of the authors and do not necessarily represent those of their affiliated organizations, or those of the publisher, the editors and the reviewers. Any product that may be evaluated in this article, or claim that may be made by its manufacturer, is not guaranteed or endorsed by the publisher.
